# Geriatric support in the emergency department: a national survey in Belgium

**DOI:** 10.1186/s12877-017-0458-8

**Published:** 2017-03-16

**Authors:** Els Devriendt, Isabelle De Brauwer, Lies Vandersaenen, Pieter Heeren, Simon Conroy, Benoit Boland, Johan Flamaing, Marc Sabbe, Koen Milisen

**Affiliations:** 1 Department of Public Health and Primary Care, Academic Centre for Nursing and Midwifery, KU Leuven, Kapucijnenvoer 35/4, 3000 Leuven, Belgium; 2 Department of Geriatric Medicine, University Hospitals Leuven, Herestraat 49, 3000 Leuven, Belgium; 3 Division of Geriatric Medicine, Cliniques Universitaires St-Luc, av Hippocrate 10, 1200 Brussels, Belgium; 4 Institute of Health and Society, Université catholique de Louvain, clos Chapelle-aux-champs 30, 1200 Brussels, Belgium; 5 Geriatric Medicine, University Hospitals of Leicester, Leicester, LE1 5WW UK; 6 Department of Clinical and Experimental Medicine, Gerontology and Geriatrics, KU Leuven, Herestraat 49, 3000 Leuven, Belgium; 7 Department of Emergency Medicine, University Hospitals Leuven, Herestraat 49, 3000 Leuven, Belgium; 8 Department of Public Health and Primary Care, Emergency Medicine, KU Leuven, Kapucijnenvoer 35/4, 3000 Leuven, Belgium

**Keywords:** Geriatric patients, Geriatric support, Emergency department, Survey

## Abstract

**Background:**

Older people in the emergency department (ED) represent a growing population and increasing proportion of the workload in the ED. This study investigated the support for frail older people in the ED, by exploring the collaboration between the geriatric services (GS) and the EDs in Belgian hospitals.

**Methods:**

An electronic cross-sectional survey in all Belgian hospitals with an ED (*n* = 100) about care aspects, collaboration, education and infrastructure for older patients in the ED was collected. Descriptive analyses were performed at national level.

**Results:**

Forty-nine of 100 surveys were completed by the GS. The heads of the ED returned only 12 incomplete questionnaires and these results are therefore not reported.

Twenty-six of the 49 heads of GSs (53%) indicated that there was an agreement, mainly informal, between the geriatric and the emergency department concerning the management of older people on the ED.

A geriatrician was available for specific problems, by phone or in person, in 96% of the EDs during daytime on weekdays. Almost all responding hospitals (96%) had an inpatient geriatric consultation team, of which 85% was available for specific problems at the ED, by phone or bedside during the daytime on weekdays. Twenty-nine heads of the GSs (59%) reported that older patients were screened at ED admission during the day to identify ‘at risk’ patients. The results of the screening were used in the context of further treatment (76%), to decide on hospital admission (27%), or to justify admission on a geriatric ward (55%). In the year preceding the survey, 25% of the responding hospitals had organised geriatric training for ED healthcare workers. Thirty-four heads of the GS (69%) felt that the infrastructure of the ED was insufficient to give high-quality care for older persons.

**Conclusion:**

Collaborations between EDs and GS are emerging in Belgium, but are currently rather limited and not yet sufficiently embedded in the ED care. Exploratory studies are necessary to identify how these collaborations can be improved.

**Electronic supplementary material:**

The online version of this article (doi:10.1186/s12877-017-0458-8) contains supplementary material, which is available to authorized users.

## Background

Older people in the emergency department (ED) represent a growing population and increasing proportion of the workload; about 12–21% of the ED patients are 65 years and older, and this number will increase [1, 2]. In 2014, 16% of the Belgian ED population was 70 years and older, 67.5% of all older people of 80 years and older was admitted after ED visit and only 30% of the older patient came without referral from the general practitioner. [3]. Two single centre cohort studies in two different hospitals in Belgium showed that older people admitted to the ED had a mean age of above 80 years old [4, 5]. The majority of these older patients’ lives at home, before ED admission and more than half had at least one fall incident in the last year. Within 3 months about 30% revisited the ED [4, 5].

International literature also shows that, older patients often present at the ED with non-specific complaints, multiple co-morbidities, physical and cognitive impairment, polypharmacy and poor social support [1, 6], rendering the care for these patients complex. Underestimation of health care problems and functional decline after ED discharge are common in older ED patients [7–9]. They are more likely to be admitted to the hospital and experience more unplanned readmissions after ED discharge compared to a younger population [7–9]. There is a need to better understand how the ED environment and care processes can be attuned to the needs of older people [1].

Recent studies have focussed on geriatric emergency care models [10–18]. These care models are characterised by different components such as geriatric risk screening, Comprehensive Geriatric Assessment (CGA), geriatric recommendations and referrals resulting in an individualised care plan and follow-up care after ED discharge. The implementation of such care models resulted in some positive trends towards service related outcomes: reductions of unplanned readmissions [11–13, 18, 19], hospitalisation [12–14, 17, 18] and patient related outcomes e.g. functional decline [15, 19, 20]. Despite the high heterogeneity in the care models, collaboration and exchange between the ED team and specialists in geriatric care is part of most models (e.g. team discussions).

In Belgium, geriatric care has been officially organised by a Royal Decree approved in 2007 [21]. This care program comprises among others an inpatient geriatric consultation team (IGCT) [22]. These mobile multidisciplinary teams support health care professionals in treating geriatric patients admitted to non-geriatric units. The main aim is to share the core geriatric principles and multidisciplinary expertise. The multidisciplinary team encompasses geriatricians, nurses specialised in geriatric care, a physiotherapist, occupational, speech and language therapist and a psychologist. At least two FTE with a maximum of 4 FTE are available per hospital, but the precise size of the staff is calculated on the annual number of older patients admitted on non-geriatric units for each hospital separately and the composition of the IGCT depends on local needs per hospital. However, presence of IGCTs on the ED is not mandatory. A Belgian survey on the implementation and performance of interdisciplinary IGCTs reported that only a minority collaborated with the ED in 2010 (11%) [23]. In response to the ageing population in Belgian hospitals and EDs, this survey aimed to investigate the current support for frail older patients in the ED, and collaborations between geriatric services and EDs in Belgian hospitals.

## Methods

### Study design

We conducted a cross-sectional survey from December 2013 till February 2014 in all Belgian hospitals with an ED (*n* = 100).

### Participants

A questionnaire was addressed to the heads of the GS and the ED. For hospitals with campuses on different geographical locations, heads of the GS and ED were asked to complete the survey for each campus separately.

### Development and validation of the questionnaire

The content of the questionnaire was developed in cooperation with the College for Geriatrics and the College for Emergency care, two bodies created by the Belgian Government to set up quality improvement initiatives in geriatric and emergency medicine, respectively.

Before finalising the questionnaire both content and face validity were evaluated. The content validity of the Dutch version of the survey was assessed by five geriatricians and one emergency physician according to the methods of Lynn and Polit (data not shown) [24, 25].

The comprehensibility and usability of the questionnaire were evaluated by five other experts from the College for Geriatrics (*n* = 4) and the College for Emergency Care (*n* = 1), by asking ‘*Is this question understandable?*’ Amendments were made to ensure clarity and ease of understanding. The Dutch questionnaire was translated in a French version by a bilingual geriatrician, but no back translation was performed.

The final questionnaire comprised 45 questions in two sections. The first section included 35 questions about the care for older patients in the ED and the collaboration within the ED (collaboration between ED physicians and geriatricians, the geriatric consultation team in the ED, accessibility of the geriatric day hospital, the use of screening tools to identify high-risk geriatric patients, geriatric education on the ED and the presence of appropriate infrastructure and specific geriatric procedures). The second section included 10 questions concerning general information of the hospital (e.g. name of the hospital, number of hospital days of people aged 75 years or older, number of geriatric beds, number of geriatricians and availability of a social worker for the ED). See Additional files 1 and 2 for the questionnaire in Dutch and French.

### Data collection

The questionnaire was sent to the heads of the GS and ED of each of the 100 hospitals. ‘Lime survey’ software was used for the on-line completion of the questionnaire. Electronic reminders were sent after 4 weeks. Participants received a 50 Euros fee after completing the questionnaire. Follow-up by phone or personal contact with possible respondents was not undertaken.

### Data analysis

Data were analysed at the aggregated, national level and not by individual hospital. Descriptive analyses were performed by using the statistical package IBM SPSS Statistics 22 (SPSS Inc., Chicago, IL).

## Results

### Sample characteristics

Of the study population (*n* = 100), 78 questionnaires were returned from the GSs. Returned questionnaires were removed if only the general information of the hospital was completed (*n* = 29). A total of 49 surveys (49% response rate) were collected successfully. Five of the seven university hospitals completed the survey, representing 10% of all respondents. The main characteristics of the sample are given in Table 1.

**Table 1:** Characteristics of the 49 responding hospitals

Region, n (%)	
Flanders	29 (59.2)
Brussels	6 (12.2)
Wallonia	14 (28.6)
Type, n (%)	
University	5 (10.2)
Non-university	44 (89.8)
Language, n (%)	
Dutch	30 (61.2)
French	19 (38.8)
Number of geriatric-beds, M ± SD (min-max)	77.3 ± 44.4 (24–244)
Number of geriatricians, M ± SD (min-max)	2.9 ± 1.6 (1–7)

*M* mean, *SD* standard deviation, *min* minimum, *max* maximum

The heads of the ED returned only 12 questionnaires. Due to the low response rate and many missing data within the returned questionnaires, the results from the ED are not further reported in this paper.

### Survey results

#### Involvement of the geriatric team in the ED

Twenty-six of the 49 heads of GSs (53%) indicated that there was an agreement, mainly informal, between the geriatric and the emergency departments concerning the management and the flow of older people in the ED (*n* = 14, 54%). Six out of 23 heads of GS without such agreements, planned to develop one in the future.

A geriatrician was available for specific problems, by phone or in person, in 96% of the EDs (*n* = 47) during the daytime on weekdays, while one third (*n* = 16) saw every older patient with a frailty profile. Five institutions held geriatric rounds in the ED at specific time points, but none provided a continuously physical presence. All these proportions decreased during the night and weekend (Table 2). Admission to a geriatric ward was agreed by a geriatrician in collaboration with an emergency physician in 43% (*n* = 21) of the hospitals, while this decision was taken by the geriatrician alone in 33% (*n* = 16). Ninety-four per cent of the heads of the GS (*n* = 46) stated that the physical availability of a geriatrician on the ED added value to the management of older people in the ED.

Almost all responding hospitals had an IGCT (96% *n* = 47) (multidisciplinary team, including the geriatrician, geriatric nurse, and in some teams paramedical caregivers e.g. occupational therapists), which 85% (*n* = 40) was available for the ED, by phone or bedside for specific problems (such as delirium and functional decline), during the daytime on weekdays. One in five of these IGCTs (*n* = 9) saw every older ED patient after a phone call from ED staff, while 9% (*n* = 4) were present in the ED at specified time-points. No IGCT was continuously present on the ED. All these proportions decreased during the night and weekend (Table 2). Forty percent (*n* = 17) of these teams used a geriatric assessment adapted to the ED context, while 42% (*n* = 18) answered to specific clinical requests. These often comprise ‘request for admission on a geriatric ward’ (74%, *n* = 32), ‘judgement about the need for hospitalisation’ (63%, *n* = 27) and the ‘application for social needs assessment’ (56%, *n* = 24). Eighty-one percent of the heads of the GS (*n* = 35) stated that the IGCT has an added value for the management of older patients in the ED. Also, 79% (*n* = 34) stated that the function of the IGCT in the ED should be extended in the future. Two thirds of the respondents who did not have an IGCT available for the ED (*n* = 4/7), clarified that an IGCT might add value to the ED by assisting in the care planning for frail older patients, assessing the need for hospitalisation and evaluating geriatric syndromes and social, functional or cognitive problems.

Data concerning availability of social workers on the ED are summarised in Table 2.

**Table 2:** Geriatrician, inpatient geriatric consultation team and social worker availability for the emergency department, by collaboration type

	Daytime, weekday			Night			Weekend		
	GER N(%) 49(100)	IGCT N(%) 47(100)	SW N(%) 49(100)	GER N(%) 49(100)	IGCT N(%) 47(100)	SW N(%) 49(100)	GER N(%) 49(100)	IGCT N(%) 47(100)	SW N(%) 49(100)
By phone	47(96)	40(85)	46(94)	27(55)	2(4)	5(10)	37(76)	5(11)	13(27)
Bedside, after phone call									
Specific cases	47(96)	43(92)	45(92)	22(45)	2(4)	2(4)	29(59)	3(6)	8(16)
Systematically	16(33)	9(19)	12(25)	2(4)	1(2)	0(0)	4(8)	1(2)	1(2)
On a specified moment	5(10)	4(9)	7(14)	4(8)	0(0)	0(0)	4(8)	0(0)	1(2)
Continuously physically present on ED	0(0)	0(0)	1(2)	0(0)	0(0)	0(0)	0(0)	0(0)	1(2)

*GER* Geriatrician, *IGCT* Inpatient Geriatric Consultation Team, *SW* Social worker

Forty-two institutions (86%) had a geriatric day hospital. The geriatric day hospital can be defined as an outpatient clinic with the objective of organising diagnostic, therapeutic and rehabilitative activities on a multidisciplinary basis [26]. Seven were located on a separate campus from the ED (16%) but most of these are available for consultation requests from all campuses (5/7).

General practitioners can often (*n* = 38; 91%) make an urgent appointment on the geriatric day hospital to avoid ED attendance. In 87% (*n* = 33) of these geriatric day hospitals, the appointment could be scheduled within three working days.

In 79% (*n* = 33) ED staff could make an urgent appointment in the geriatric day hospital to avoid hospitalisation. In 82% (*n* = 27) of the cases, this was possible within three working days.

#### Geriatric screening to identify high-risk geriatric patients

Twenty-nine heads of the GS (59%) reported that older patients were screened at ED admission during the day to identify ‘at risk’ patients. However, during night and weekend this decreased to 45% (*n* = 22) and 49% (*n* = 24), respectively.

The screening tools used were the ‘Identification of Seniors At Risk’ (ISAR) (*n* = 16) and the ‘Flemish Triage Risk Screening Tool’ (fTRST) (*n* = 12) [27, 28]. One customised (or locally adapted) screening tool was mentioned.

The screening was conducted by an ED nurse in 24 hospitals (83%), by the IGCT in four (14%) or by the emergency physician in one (3%). Half of the screening results were not consistently noted in the patient record (*n* = 16).

The screening results assisted health care providers in their decision to call for geriatric support. This is the IGCT in 13 EDs (59.1%) or the geriatrician in six EDs (27%). Furthermore, screening results were used in the context of further treatment (76%, *n* = 22), to decide a hospital admission (27%, *n* = 6), or to support admission on a geriatric ward (55%, *n* = 12).

More than half of the head geriatricians indicated that patients screened as ‘high-risk’ on the ED, should be evaluated by a member of the geriatric team or might benefit from a short-term referral to the GS (geriatric ward or outpatient clinic) before hospital discharge (*n* = 17).

#### Geriatric education

During the past year, 25% (*n* = 12) of the responding hospitals organised a geriatric training for the ED healthcare workers. The mean geriatric training time was 2.9 h (SD = 2.2) per year.

Training topics were subdivided into two categories: medical problems (e.g. cardiac problems in older people) and management of frail older patients in the ED (e.g. characteristics of a frail older patient).

#### ED Infrastructure

Thirty-four heads of the GS (69%) felt that the ED infrastructure was insufficient to give high-quality care for older people (score 0–10, insufficient = score 5/10 or lower). Most of them (*n* = 30, 61%) scored ≤ 5/10 for the suitability of ED infrastructure to address to the complex needs of older persons. Only nine (18%) gave a score ≥7/10.

## Discussion

Management of older people in the ED is an important issue, not only because of their rapidly increasing proportion, but importantly because of their specific and complex characteristics and risk of adverse outcomes. This survey aimed to evaluate existing support for geriatric patients in Belgian EDs, including infrastructure and education issues, and to explore perspectives on collaboration between GS and ED.

Belgian GSs are present in most EDs, however, this presence varied substantially. Geriatric support in the ED was mainly available on-call, for specific problems and during daytime and weekdays. The availability of IGCTs for the ED in 2014 was 85%, which is much higher than the 11% reported in 2010 in a previous Belgian survey [23]. In 2014, only a few GSs provided systematic support, and none offered a continuous presence in the ED. This might introduce important variation in care quality as ED patients are present 24 h per day, 7 days a week. The heads of GS were however convinced about the added-value of geriatric interventions in ED and argued that geriatric support in ED should be extended in the future. One explanation for the variation in the presence of the GSs in the ED can be the lack of strict conditions in the Royal decree (Belgian Care Program for Older People). The availability of the team, the local model of geriatric care and the pragmatic organisation (schedule) are hospital-dependant.

The most frequent reason to consult a geriatric team in the ED was a hospitalisation request. Involving a geriatric care team in ED discharge planning can lead to avoidance of hospital admissions and ED readmissions, more referrals to outpatient services, and more appropriate allocation to geriatric wards [29–31]. Proactive screening for frailty is also important as it can alert emergency caregivers about potential non-urgent geriatric problems, which need further assessment. Despite screening for frailty in all older inpatients being mandatory since 2007 in Belgium, the present survey revealed that it is not widely performed nor documented in the ED notes. Screening is mostly postponed to a later moment during admission. Furthermore, one in four hospitals did not use this frailty profile in the care process of the older people attending the ED. As a first step, screening tools could serve as “triggers” to identify the need for further geriatric assessment. Also, literature suggests that screening in the ED improves efficiency of geriatric interventions [30, 32–34]. Screening in ED could help in bed allocation for patients requiring hospitalisation, assigning the most vulnerable to geriatric evaluation and management units, where patient outcomes are better [35]. Secondly screening might sensitise the healthcare workers on the ED with regard to the needs of frail older people.

As many professionals are involved in the unplanned care for older people admitted to the ED, knowledge and expertise is needed to deal with the needs of these patients.

Geriatric education is frequently lacking in Belgian EDs, according to this study (mean 2.9 h per year), with only one in four hospitals having organised geriatric training for the ED in the previous year. Collaboration between the ED and a geriatric expert team can support care delivery, education and training [36, 37]. The geriatric heads also reported that ED infrastructure could be improved to meet the specific needs of the older patients - a finding which is consistent with literature [38, 39]. Inadequate infrastructure could expose vulnerable patients to adverse events, e.g. the development of pressure sores when waiting long time on a stretcher. Some studies report that emergency caregivers are concerned by the challenge of managing older patients with complex care needs, and that they would like to receive more training in the concept of senior friendly emergency medicine [40, 41]. Prendergasts’ study demonstrated that a hands-on approach is the preferred learning model for emergency caregivers [41]. Geriatric consultation teams could play an important educational role, by disseminating geriatric knowledge (including in the basic care needs of older people) and being role models in the ED.

The reported increase of geriatric involvement on the ED aligns with the idea of rethinking structures and processes to optimise care for vulnerable older adults. In order to do so one of the main things is the availability of data on ED visits by older patients. Indeed, having good available data systems is important to be able to understand the clinical context and monitor changes with interventions for improving hospital systems for older people. Currently the Belgian government explores which health care information systems are most suitable (e.g. such as the inter-RAI instruments - resident assessment instruments). These systems give an overview of the care profile of older people in the different settings and have, besides assistance to the clinical practice, also the aim to support policy decisions.

As the classic ED model focuses on rapid assessment and referral; an evolution towards a geriatric ED care model, that addresses complexities among older patients, seems desirable. Providing continuity of care after the ED visit is an essential component of these initiatives, for both inpatients and patients discharged [29, 31, 42, 43]. For this purpose, a follow-up can be planned in a geriatric day hospital. This study demonstrated that already most of the surveyed hospitals allow a referral by the ED or by the general practitioner to the geriatric day hospital. Rapid geriatric evaluation in these outpatient clinics might prevent some of the frequent reported emergency (re)admission(s). General practitioners and Emergency physicians should be better informed about the assessment and care possibilities in geriatric day hospitals to avoid parallel emergency visits to day hospitals overwhelming the limited geriatric resources. GS have to take responsibility to ensure that general practitioners as well as ED caregivers are well informed about how to refer and what the criteria are. Again, it is important to have uniform data systems over the different settings to improve information transfer and coordination of care. Health care information systems (such as the above mentioned inter-RAI instruments - resident assessment instruments) can play an important role.

The main limitation of this study is that analysis of the ED physicians’ views was not feasible due to non-response or many missing data. The reason for this low response is related to some issues apart from this study, and as a consequence limits the generalisation and confirmation of the results. The exact reasons – as indicated by the Belgian college for emergency care - for the low response is twofold. On the one hand, at the moment of the survey, a lot of authorities asked co-operation from the college. This forced the college to choose and set priorities. Our survey about the collaboration with the geriatricians was at that moment, for fear of overburden to the emergency departments, not the first research priority. On the other hand, the college for emergency care had some negative experiences in the collaboration with other surveys in the past; withholding them to fully participate in our survey. However, during several informal contacts between the researchers and several ED physicians, the latter confirmed the results, indicating that views of ED physicians might correspond to those of the geriatric department. Although the questionnaire was addressed by email, with an electronic reminder, to all Belgian GS heads in their main language (Dutch or French), the response rate of the survey was moderate (49%). The GS heads who returned a complete survey may have different practices and opinions on EDs than the ones who did not respond. This strengthens our conclusions that collaboration between EDs and GDs is emerging in Belgium, but it is currently rather limited and not yet sufficiently embedded in the ED care. The response rate was similar in the three different regions of Belgium (Flanders, Brussels, and Wallonia). Five of the seven university hospitals participated and so academic centres may have been over-represented. As university hospitals often have more resources and another organisation of care, readers should consider possible overrepresentation of innovative care models.

Next, in most Belgian hospitals social workers on the ED are in charge of discharge planning and psychosocial support of patients on the ED. Unfortunately, this survey did not inquire about the social worker and as a consequence we cannot evaluate its specific role in relation to the IGCT and geriatricians on the ED.

Finally, a newly developed questionnaire was used. Although its content and face validity was evaluated by experts, lack of testing other psychometric properties, i.e. inter and intra-reliability, could have affected the results. Nonetheless this study has the advantage to highlight some key aspects on the current collaboration between ED and GS in the management of older people in Belgium.

## Conclusion

In conclusion, some collaboration between ED and GS is emerging in Belgium, but is currently rather limited and not yet sufficiently embedded in the ED care. Geriatric teams are aware of the added-value of geriatric interventions in ED (i.e. by geriatricians or geriatric consultation teams) and argued that a geriatric function should be embedded in the ED. Based on these results, the authors advise that future geriatric interventions on the ED should include the identification of frail older people, as a first step, in order to optimise the use of resources. Second an adapted geriatric assessment for these patients can be useful for the identification of geriatric problems. Third, linking the findings of the geriatric assessment to a further trajectory for the patients in- or outside the hospital and giving advice to healthcare workers to improve the care for older patients is also a key component for improving geriatric care at the ED. Fourth, follow-up after the ED visit to implement and adjust advices is necessary either at home and in the hospital. Finally, sensitization and education of all healthcare workers involved remains important for the success of a qualitative approach of older people admitted to the ED.

Studies with specific attention to macro-level concerns (e.g. political framework of the countries) and insights in data on macro level are necessary to explore how collaboration can be improved.

## Additional files

Additional file 1: Questionnaire geriatric support in the emergency department: Dutch version of the questionnaire for geriatric department and emergency department. (DOCX 44 kb)
Additional file 2: Questionnaire geriatric support in the emergency department: French version of the questionnaire for geriatric department and emergency department. (DOCX 44 kb)

## Abbreviations

CGA: Comprehensive geriatric assessment
ED: Emergency Department
GS: Geriatric service
IGCT: Inpatient geriatric consultation team
ISAR: Identification of Seniors At Risk
TRST: Triage Risk Screening Tool

## References

[1] Aminzadeh F, Dalziel WB (2002). Older adults in the emergency department: a systematic review of patterns of use, adverse outcomes, and effectiveness of interventions. Ann Emerg Med.

[2] Samaras N, Chevalley T, Samaras D, Gold G (2010). Older patients in the emergency department: a review. Ann Emerg Med.

[3] Directoraat-Generaal Gezondheidszorg FV, Veiligheid van de Voedselketen en Leefmilieu. : MZG-registratie beheerd door de dienst datamanagement. 2014.

[4] De Brauwer I, D’Hoore W, Swine C, Thys F, Beguin C, Cornette P (2014). Changes in the clinical features of older patients admitted from the emergency department. Arch Gerontol Geriatr.

[5] Deschodt M, Devriendt E, Sabbe M, Gillet JB, Knockaert D, Boonen S, Flamaing J, Milisen K, Correspondence A, M. Deschodt HSaNRKULB: Older adults admitted to the emergency department (ED): Risk factors for unplanned ED readmission. *Eur Geriatr Med* 2013, 4 SUPPL. 1:S86-S87.

[6] Salvi F, Morichi V, Grilli A, Giorgi R, De Tommaso G, Dessi-Fulgheri P (2007). The elderly in the emergency department: a critical review of problems and solutions. Intern Emerg Med.

[7] Gray LC, Peel NM, Costa AP, Burkett E, Dey AB, Jonsson PV, Lakhan P, Ljunggren G, Sjostrand F, Swoboda W (2013). Profiles of older patients in the emergency department: findings from the interRAI Multinational Emergency Department Study. Ann Emerg Med.

[8] Schrijver EJ, Toppinga Q, de Vries OJ, Kramer MH, Nanayakkara PW (2013). An observational cohort study on geriatric patient profile in an emergency department in the Netherlands. Neth J Med.

[9] Schnitker L, Martin-Khan M, Beattie E, Gray L (2011). Negative health outcomes and adverse events in older people attending emergency departments: A systematic review. Australas Emerg Nurs J.

[10] Conroy SP, Stevens T, Parker SG, Gladman JR (2011). A systematic review of comprehensive geriatric assessment to improve outcomes for frail older people being rapidly discharged from acute hospital: ‘interface geriatrics’. Age Ageing.

[11] Foo CL, Siu VW, Tan TL, Ding YY, Seow E (2012). Geriatric assessment and intervention in an emergency department observation unit reduced re-attendance and hospitalisation rates. Australas J Ageing.

[12] Arendts G, Fitzhardinge S, Pronk K, Donaldson M, Hutton M, Nagree Y (2012). The impact of early emergency department allied health intervention on admission rates in older people: a non-randomized clinical study. BMC Geriatr.

[13] Arendts G, Fitzhardinge S, Pronk K, Hutton M (2013). Outcomes in older patients requiring comprehensive allied health care prior to discharge from the emergency department. Emerg Med Australas.

[14] Wright PN, Tan G, Iliffe S, Lee D (2014). The impact of a new emergency admission avoidance system for older people on length of stay and same-day discharges. Age Ageing.

[15] Foo CL, Siu VW, Ang H, Phuah MW, Ooi CK (2014). Risk stratification and rapid geriatric screening in an emergency department - a quasi-randomised controlled trial. BMC Geriatr.

[16] Ellis G, Jamieson CA, Alcorn M, Devlin V (2012). An Acute Care for Elders (ACE) unit in the emergency department. Eur Geriatr Med.

[17] Keyes DC, Singal B, Kropf CW, Fisk A (2014). Impact of a new senior emergency department on emergency department recidivism, rate of hospital admission, and hospital length of stay. Ann Emerg Med.

[18] Conroy SP, Ansari K, Williams M, Laithwaite E, Teasdale B, Dawson J, Mason S, Banerjee J (2014). A controlled evaluation of comprehensive geriatric assessment in the emergency department: the ‘Emergency Frailty Unit’. Age Ageing.

[19] Caplan GA, Williams AJ, Daly B, Abraham K (2004). A randomized, controlled trial of comprehensive geriatric assessment and multidisciplinary intervention after discharge of elderly from the emergency department--the DEED II study. J Am Geriatr Soc.

[20] McCusker J, Verdon J, Tousignant P, de Courval LP, Dendukuri N, Belzile E (2001). Rapid emergency department intervention for older people reduces risk of functional decline: results of a multicenter randomized trial. J Am Geriatr Soc.

[21] Baeyens JP (2010). Belgian care programme for older patients. J Nutr Health Aging.

[22] Braes T, Flamaing J, Pelemans W, Milisen K (2009). Geriatrics on the run: rationale, implementation, and preliminary findings of a Belgian internal liaison team. Acta Clin Belg.

[23] Deschodt M, Flamaing J, Rock G, Boland B, Boonen S, Milisen K (2012). Implementation of inpatient geriatric consultation teams and geriatric resource nurses in acute hospitals: a national survey study. Int J Nurs Stud.

[24] Polit DF, Beck CT, Owen SV (2007). Is the CVI an acceptable indicator of content validity? Appraisal and recommendations. Res Nurs Health.

[25] Lynn MR (1986). Determination and quantification of content validity. Nurs Res.

[26] Velghe A, Kohn L, Petermans J, Gillain D, Petrovic M, Van Den Noortgate N (2011). The Belgian geriatric day hospitals as part of a care program for the geriatric patient: first results of the implementation at the national level. Acta Clin Belg.

[27] McCusker J, Bellavance F, Cardin S, Trepanier S (1998). Screening for geriatric problems in the emergency department: reliability and validity. Identification of Seniors at Risk (ISAR) Steering Committee. Acad Emerg Med.

[28] Moons P, De Ridder K, Geyskens K, Sabbe M, Braes T, Flamaing J, Milisen K (2007). Screening for risk of readmission of patients aged 65 years and above after discharge from the emergency department: predictive value of four instruments. Eur J Emerg Med.

[29] Hegney D, Buikstra E, Chamberlain C, March J, McKay M, Cope G, Fallon T (2006). Nurse discharge planning in the emergency department: a Toowoomba, Australia, study. J Clin Nurs.

[30] Sinha SK, Bessman ES, Flomenbaum N, Leff B (2011). A systematic review and qualitative analysis to inform the development of a new emergency department-based geriatric case management model. Ann Emerg Med.

[31] Ballabio C, Bergamaschini L, Mauri S, Baroni E, Ferretti M, Bilotta C, Vergani C (2008). A comprehensive evaluation of elderly people discharged from an Emergency Department. Intern Emerg Med.

[32] Fealy G, McCarron M, O’Neill D, McCallion P, Clarke M, Small V, O’Driscoll A, Cullen A (2009). Effectiveness of gerontologically informed nursing assessment and referral interventions for older persons attending the emergency department: systematic review. J Adv Nurs.

[33] Graf CE, Zekry D, Giannelli S, Michel JP, Chevalley T (2010). Comprehensive Geriatric Assessment in the Emergency Department. J Am Geriatr Soc.

[34] Hastings SN, Heflin MT (2005). A systematic review of interventions to improve outcomes for elders discharged from the emergency department. Acad Emerg Med.

[35] Ellis G, Whitehead MA, Robinson D, O’Neill D, Langhorne P (2011). Comprehensive geriatric assessment for older adults admitted to hospital: meta-analysis of randomised controlled trials. BMJ.

[36] Ellis G, Marshall T, Ritchie C (2014). Comprehensive geriatric assessment in the emergency department. Clin Interv Aging.

[37] Carpenter CR, Griffey RT, Stark S, Coopersmith CM, Gage BF (2011). Physician and nurse acceptance of technicians to screen for geriatric syndromes in the emergency department. Western J Emerg Med.

[38] Hwang U, Morrison RS (2007). The geriatric emergency department. J Am Geriatr Soc.

[39] Shanley C, Sutherland S, Stott K, Tumeth R, Whitmore E (2008). Increasing the profile of the care of the older person in the ED: a contemporary nursing challenge. Int Emerg Nurs.

[40] Schumacher JG, Deimling GT, Meldon S, Woolard B (2006). Older adults in the Emergency Department: predicting physicians’ burden levels. J Emerg Med.

[41] Prendergast HM, Jurivich D, Edison M, Bunney EB, Williams J, Schlichting A (2010). Preparing the front line for the increase in the aging population: geriatric curriculum development for an emergency medicine residency program. J Emerg Med.

[42] Guttman A, Afilalo M, Guttman R, Colacone A, Robitaille C, Lang E, Rosenthal S (2004). An emergency department-based nurse discharge coordinator for elder patients: does it make a difference?. Acad Emerg Med.

[43] Rosted E, Poulsen I, Hendriksen C, Petersen J, Wagner L (2013). Testing a two step nursing intervention focused on decreasing rehospitalizations and nursing home admission post discharge from acute care. Geriatr Nurs (New York, NY).

