# Supplementary material for: Geriatric support in the emergency department: a national survey in Belgium
Source: BMC Geriatr. 2017 Mar 16;17:68. doi: 10.1186/s12877-017-0458-8 (PMC5356306; doi:10.1186/s12877-017-0458-8)
Supplement: Additional file 1: — Questionnaire geriatric support in the emergency department: Dutch version of the questionnaire for geriatric department and emergency department. (DOCX 44 kb) [file 12877_2017_458_MOESM1_ESM.docx]

**Appendix 1: Questionnaire geriatric support in the emergency department :Dutch version of the questionnaire for geriatric department and emergency department**

Geriatrische zorg voor ouderen op spoedgevallen: een nationale survey (diensthoofd geriatrie)

**Deel 1:**

*inhoudelijke vragen over de zorg voor ouderen* op en in samenwerking met de dienst spoedgevallen

1. Er zijn **formele afspraken*** tussen huisartsen in de regio en het ziekenhuis met betrekking tot de verwijzing van ouderen via de dienst spoedgevallen.

- Ja (gelieve protocol mee te sturen)
- Neen

* def formele afspraken: geschreven afspraken door beide partijen (ziekenhuizen en huisartsen) erkend en gecommuniceerd naar de volledige huisartsenwachtkring binnen de regio.

1. Ga na op welke manier de 10 ouderen die zich recent aanmelden op uw spoedgevallen werden doorverwezen. Op welke wijze werden de tien laatste ouderen van 75 jaar of ouder doorverwezen naar uw dienst spoedgevallen?

Info: Neem als referentie, een gewone weekdag overdag

Info: Er zijn meerdere antwoordcategorieën mogelijk. Gelieve bij elke antwoordcategorie het desbetreffende aantal patiënten aan te duiden (0 = geen patiënten).

- Spontane aanmelding: …. patiënten
- Hulpcentrum 100: …patiënten
- Verwijsbrief: …patiënten
- Telefonisch contact met de urgentiearts of geriater: …patiënten
- Andere, specifieer...

1. Welke methode van afspraken verkiest u bij opname van een oudere via de dienst spoedgevallen?

- Informele afspraken: verwijsbrief
- Informele afspraken: telefonisch contact met geriater of urgentiearts
- Informele afspraken: verwijsbrief in combinatie met telefonische afspraken
- Formele afspraken binnen de regio

1. Beschikt uw ziekenhuis over een **dagziekenhuis geriatrie**?

- Ja
- Neen, ga dan onmiddellijk naar vraag 6

1. Kan vanuit de dienst spoedgevallen een dringende afspraak gemaakt worden op het **dagziekenhuis geriatrie** om een hospitalisatie te vermijden?

- Neen, dat kan niet
- Ja, dat kan,
  - Binnen één werkdag
  - Binnen één tot drie werkdagen
  - Binnen langer dan drie werkdagen

1. Bestaat er voor uw ziekenhuis een **procedure** (bijvoorbeeld een klinisch zorgpad) tussen geriatrie en spoedgevallen?

- Neen, er bestaat geen procedure
- Ja, er bestaat een formele, uitgeschreven procedure (gelieve dit protocol mee te sturen)
- Ja, er bestaat een informele procedure die niet uitgeschreven is
- Momenteel bestaat er geen procedure, maar er zijn plannen voor de toekomst om dit uit te werken

1. Beschikt uw **ziekenhuis** over een intern liaison team geriatrie*?

- Ja
- Neen, ga dan onmiddellijk naar vraag 14

* De term intern geriatrisch liaison team heeft ook synoniemen bijvoorbeeld geriatrisch consultatie team, geriatrisch support team, … In het verder verloop van deze vragenlijst wordt de benaming intern liaison team geriatrie afgekort naar ILT geriatrie.

1. Gelieve in onderstaande tabel per stelling de beschikbaarheid **van het ILT geriatrie** op de dienst spoedgevallen voor de verschillende tijdsmomenten (dag, nacht, weekend) aan te duiden

|  | Dag | Nacht | Weekend |
| --- | --- | --- | --- |
| Een **medewerker van het intern liaisonteam geriatrie** is telefonisch consulteerbaar voor de oudere patiënt op spoedgevallen. | - Ja - Neen | - Ja - Neen | - Ja - Neen |
| Een **medewerker van het intern liaisonteam geriatrie** is na telefonisch contact beschikbaar aan het bed van de oudere patiënt op spoedgevallen voor specifieke casussen | - Ja - Neen | - Ja - Neen | - Ja - Neen |
| Een **medewerker van het intern liaisonteam geriatrie** komt na telefonisch contact systematisch aan het bed van de oudere patiënt op spoedgevallen voor alle casussen. | - Ja - Neen | - Ja - Neen | - Ja - Neen |
| Een **medewerker van het intern liaisonteam geriatrie** is op afgesproken momenten aanwezig op spoedgevallen. | - Ja - Neen | - Ja - Neen | - Ja - Neen |
| **Een medewerker van het intern liaisonteam geriatrie** is continu aanwezig op spoedgevallen | - Ja - Neen | - Ja - Neen | - Ja - Neen |

Indien overal neen geantwoord, ga naar vraag 14

1. In welke situaties wordt voornamelijk gebruik gemaakt van h**et ILT geriatrie** op de dienst spoedgevallen? Gelieve hieronder de 3 meest voorkomende situaties in uw ziekenhuis aan te duiden.

- Twijfel over de nood aan hospitalisatie
- Vraag op-/overname geriatrie
- Functionele problematiek
- Cognitieve problematiek
- Nood aan medische expertise
- Nood aan verdere diagnostiek
- Nood aan oppuntstelling van de sociale situatie (evaluatie en aanpassing van de thuiszorg)
- Andere, specificeer: …..

1. Hoeveel keer per maand wordt het **ILT geriatrie** geconsulteerd door de dienst spoedgevallen? …. Oproepen/ maand
2. Het ILT geriatrie dat langskomt op spoedgevallen zal:

- Uitsluitend een antwoord bieden op de consultvraag
- Een basis assessment uitvoeren aangepast voor de dienst spoedgevallen
- Een standaard assessment uitvoeren op spoedgevallen net als op de andere hospitalisatiediensten

1. Beoordeel volgende stelling: De bestaande werking van het **ILT geriatrie** op spoedgevallen moet verder uitgebreid worden.

○ Helemaal niet akkoord ○ Eerder niet akkoord ○ Eerder akkoord ○ Helemaal akkoord

1. Beoordeel volgende stelling: Het **ILT geriatrie** is een meerwaarde op de dienst spoedgevallen.

○ Helemaal niet akkoord ○ Eerder niet akkoord ○ Eerder akkoord ○ Helemaal akkoord

1. Beoordeel volgende stelling: Het **ILT geriatrie** zou een meerwaarde zijn op de dienst spoedgevallen. (enkel in te vullen indien neen op vraag 7 of 8)

○ Helemaal niet akkoord ○ Eerder niet akkoord ○ Eerder akkoord ○ Helemaal akkoord

1. Beoordeel volgende stelling: De fysieke beschikbaarheid van een erkend specialist **geriatrie** op de dienst spoedgevallen is een meerwaarde.

○ Helemaal niet akkoord ○ Eerder niet akkoord ○ Eerder akkoord ○ Helemaal akkoord

1. Gelieve in onderstaande tabel elke stelling voor de verschillende tijdsmomenten (dag, nacht, weekend) te beoordelen.

|  | Dag | Nacht | Weekend |
| --- | --- | --- | --- |
| Bij opname op de spoedgevallen van uw ziekenhuis wordt doorgaans een **urgentie triage** toegepast die internationaal erkend is (vb Manchester, ESI,...) | - Ja - Neen | - Ja - Neen | - Ja - Neen |
| Tijdens de opname op de spoedgevallen van uw ziekenhuis wordt voor ouderen een **screeningsinstrument** gebruikt om patiënten met een geriatrisch risicoprofiel in kaart te brengen (vb ISAR, VIP, TRST,...) (indien neen, ga naar vraag 22) | - Ja - Neen | - Ja - Neen | - Ja - Neen |

1. Welk screeningsinstrument wordt gebruikt om patiënten met een geriatrisch risicoprofiel in kaart te brengen op de spoedgevallen van uw ziekenhuis

- Niet huisgemaakt screeningsinstrument: …..
- Huisgemaakt screeningsinstrument: …

1. Welke Cutoff score wordt gehanteerd? …..
2. Wie vult het screeningsinstrument om patiënten met een geriatrisch risicoprofiel in kaart te brengen in op spoedgevallen van uw ziekenhuis?

- Spoedgevallen verpleegkundige
- Urgentie arts
- Intern geriatrisch liaison team
- Andere, namelijk:

1. Wordt het resultaat van het screeningsinstrument om patiënten met een geriatrisch risicoprofiel in kaart te brengen op de spoedgevallen genoteerd in het patiëntendossier?

- Ja, altijd
- Ja, soms
- Neen

1. Worden de resultaten van het screeningsinstrument om patiënten met een geriatrisch risicoprofiel in kaart te brengen gebruikt in het kader van de verdere behandeling (zowel tijdens als na het verblijf op spoedgevallen) van de patiënt?

- Neen, de resultaten worden niet verder gebruikt
- Ja, de resultaten (meerdere antwoord categorieën zijn mogelijk):
  - Ondersteunen de zorgverleners op spoedgevallen in de beslissing om al dan niet het intern geriatrisch liaison team in te schakelen
  - Ondersteunen de zorgverleners op spoedgevallen in de beslissing om al dan niet een geriater in te schakelen
- Ondersteunen de zorgverleners op spoedgevallen in de beslissing om een patiënt al dan niet op de dienst geriatrie te hospitaliseren
- Ondersteunen de zorgverleners op spoedgevallen in de beslissing om de patiënt al dan niet te hospitaliseren of naar huis te ontslaan
- Andere, specificeer: ………………………………………………………

1. Moet een patiënt met een geriatrisch risicoprofiel (positieve screening), die na diagnosestelling en behandeling op spoedgevallen terug naar huis ontslagen wordt, voor ontslag systematisch gezien worden / doorverwezen worden naar een medewerker van **de dienst geriatrie?**

- Neen
- Ja

1. Zijn er in het jaar 2012-2013 opleidingsinitiatieven geweest van de dienst geriatrie specifiek voor de dienst spoedgevallen?

- Neen
- Ja

1. Specifieer het totaal aantal uren van de opleidingsinitiatieven:….
2. Specifieer de datum van het laatste opleidingsinitiatief:
3. Specifieer het onderwerp van het laatste opleidingsinitiatief:
4. Beoordeel volgende stelling: Op de dienst spoedgevallen is de infrastructuur voldoende uitgerust voor de kwaliteitsvolle opvang van ouderen. Vb architectuur, hulpmiddelen

○ Helemaal niet akkoord ○ Eerder niet akkoord ○ Eerder akkoord ○ Helemaal akkoord

1. Heeft uw ziekenhuis reeds speciale initiatieven die hierboven nog niet vermeld werden, genomen om de zorg voor ouderen op spoedgevallen te verbeteren?

- Neen
- Ja, specifieer

1. In welke mate is de zorg voor ouderen op uw spoedgevallendienst aangepast aan de specifieke zorgnoden van ouderen? Beoordeel op een schaal van 1 tot 10 (1 = slecht, 10 = zeer goed).

○ 0 ○1 ○2 ○3 ○4 ○5 ○6 ○7 ○8 ○9 ○10

**Deel 2 Algemene ziekenhuisgegevens**

1. Wat is de naam van uw (fusie)ziekenhuis? …….
2. Wat is het erkenningsnummer van uw (fusie)ziekenhuis? …..
3. Gelieve onderstaande tabel voor uw ziekenhuis aan te vullen. Er is in de tabel één lijn per campus voorzien. Met andere woorden indien uw ziekenhuis slechts over 1 campus beschikt dient u slechts één lijn in te vullen, zijn er meerdere campussen dan vult u een evenredig aantal lijnen in.

| Naam campus | Spoedgevallendienst op de campus | Aantal benutte G-bedden op de campus* | Dagziekenhuis geriatrie op de campus | Intern geriatrisch liaisonteam op de campus |
| --- | --- | --- | --- | --- |
|  | - Ja, dienst spoedgevallen met functie ‘gespecialiseerde spoedgevallenzorg’ - Ja, dienst spoedgevallen met functie ‘eerste opvang spoedgevallen’ - Neen |  | - Ja - Neen | - Ja - Neen |
| Gelieve de gegevens van de verschillende campussen op dezelfde manier aan te vullen op de website | | | | |

* def benutte G-bedden: hiermee bedoelen we het aantal G-bedden die momenteel gebruikt worden voor de hospitalisatie van geriatrische patiënten. Dit aantal kan overeen komen met het aantal G-bedden waarvoor u ziekenhuis een erkenning heeft, maar kan ook een ander getal zijn indien niet alle erkende bedden gebruikt worden.

1. Gelieve onderstaande tabel voor uw ziekenhuis aan te vullen.

*U dient één lijn per geriater in te vullen. Bijvoorbeeld: er zijn drie geriaters in uw ziekenhuis tewerkgesteld, gelieve dan ook drie lijnen in te vullen.

| Geriater | Totale tewerkstelling onder contract met het ziekenhuis (in 10de VTE, met uitzondering van de 10de "academische tewerkstelling") (.../10) | Tewerkgesteld op de dienst geriatrie (in 10de VTE) (.../10) | Werkzaam op de campus met dienst spoedgevallen (ja/neen) | Tewerkgesteld op de dienst spoedgevallen (in 10de VTE) (.../10) |
| --- | --- | --- | --- | --- |
| Geriater | … /10 | … /10 | - Ja - Neen | … /10 |
| Gelieve de gegevens van de verschillende geriaters op dezelfde manier aan te vullen op de website | | | | |

* Gelieve bij de totale tewerkstelling en de tewerkstelling op de dienst geriatrie/spoedgevallen een getal tussen 0 en 10 in te vullen. Gelieve bij de werkzaamheid op de campus met dienst spoedgevallen 'ja' of 'neen' in te vullen.

1. Gelieve in onderstaande tabel per stelling de beschikbaarheid van **een erkend specialist geriatrie (geen ASO)** op de dienst spoedgevallen voor de verschillende tijdsmomenten (dag, nacht, weekend) aan te duiden.

|  | Dag | Nacht | Weekend |
| --- | --- | --- | --- |
| Een **geriater** is telefonisch consulteerbaar voor de oudere patiënt op spoedgevallen | - Ja - Neen | - Ja - Neen | - Ja - Neen |
| Een **geriater** is na telefonisch contact beschikbaar aan het bed van de oudere patiënt op spoedgevallen voor specifieke casussen | - Ja - Neen | - Ja - Neen | - Ja - Neen |
| Een **geriater** komt na telefonisch contact systematisch aan het bed van de oudere patiënt op spoedgevallen voor alle casussen. | - Ja - Neen | - Ja - Neen | - Ja - Neen |
| Een **geriater** is op afgesproken momenten aanwezig op spoedgevallen | - Ja - Neen | - Ja - Neen | - Ja - Neen |
| Een **geriater** is continu aanwezig op spoedgevallen | - Ja - Neen | - Ja - Neen | - Ja - Neen |

1. Welke **discipline** neemt doorgaans de **finale beslissing** om een oudere patiënt van op spoedgevallen te **hospitaliseren op de dienst geriatrie**?

- De geriater
- De urgentiearts
- Beide partijen na overleg
- Andere, specificeer: …..

1. Gelieve in onderstaande tabel per stelling de beschikbaarheid van **de sociale dienst** op de dienst spoedgevallen voor de verschillende tijdsmomenten (dag, nacht, weekend) aan te duiden.

|  | Dag | Nacht | Weekend |
| --- | --- | --- | --- |
| De **sociale dienst** is telefonisch consulteerbaar voor de oudere patiënt op spoedgevallen | - Ja - Neen | - Ja   Neen | - Ja   Neen |
| De **sociale dienst** is na telefonisch contact beschikbaar aan het bed van de oudere patiënt op spoedgevallen voor specifieke casussen | - Ja - Neen | - Ja - Neen | - Ja - Neen |
| De **sociale dienst** komt na telefonisch contact systematisch aan het bed van de oudere patiënt op spoedgevallen voor alle casussen. | - Ja - Neen | - Ja - Neen | - Ja - Neen |
| De **sociale dienst** is op afgesproken momenten aanwezig op spoedgevallen | - Ja - Neen | - Ja - Neen | - Ja - Neen |
| De **sociale dienst** is continu aanwezig op spoedgevallen | - Ja - Neen | - Ja - Neen | - Ja - Neen |

1. Gelieve hieronder eventuele aanvullende opmerkingen/ suggesties over de zorg voor ouderen op spoedgevallen te noteren
2. Indien we bijkomende vragen hebben, op welk telefoonnummer of e-mailadres kunnen we u het beste bereiken?

Naam: …

Telefoonnummer: …

E-mail adres: …

1. Gelieve hier uw bankrekeningnummer achter te laten

……………………………………………………..

Naam: …………………………

**Hartelijk dank voor uw waardevolle bijdrage!**

Geriatrische zorg voor ouderen op spoedgevallen: een nationale survey (diensthoofd spoedgevallen)

**Deel 1:**

*inhoudelijke vragen over de zorg voor ouderen* op en in samenwerking met de dienst spoedgevallen

1. Er zijn **formele afspraken*** tussen huisartsen in de regio en het ziekenhuis met betrekking tot de verwijzing van ouderen via de dienst spoedgevallen.

- Ja (gelieve protocol mee te sturen)
- Neen

* def formele afspraken: geschreven afspraken door beide partijen (ziekenhuizen en huisartsen) erkend en gecommuniceerd naar de volledige huisartsenwachtkring binnen de regio.

1. Ga na op welke manier de 10 ouderen die zich recent aanmelden op uw spoedgevallen werden doorverwezen. Op welke wijze werden de tien laatste ouderen van 75 jaar of ouder doorverwezen naar uw dienst spoedgevallen?

Info: Neem als referentie, een gewone weekdag overdag

Info: Er zijn meerdere antwoordcategorieën mogelijk. Gelieve bij elke antwoordcategorie het desbetreffende aantal patiënten aan te duiden (0 = geen patiënten).

- Spontane aanmelding: …. patiënten
- Hulpcentrum 100: …patiënten
- Verwijsbrief: …patiënten
- Telefonisch contact met de urgentiearts of geriater: …patiënten
- Andere, specifieer...

1. Welke methode van afspraken verkiest u bij opname van een oudere via de dienst spoedgevallen?

- Informele afspraken: verwijsbrief
- Informele afspraken: telefonisch contact met geriater of urgentiearts
- Informele afspraken: verwijsbrief in combinatie met telefonische afspraken
- Formele afspraken binnen de regio

1. Beschikt uw ziekenhuis over een **dagziekenhuis geriatrie**?

- Ja
- Neen, ga dan onmiddellijk naar vraag 6

1. Kan vanuit de dienst spoedgevallen een dringende afspraak gemaakt worden op het **dagziekenhuis geriatrie** om een hospitalisatie te vermijden?

- Neen, dat kan niet
- Ja, dat kan,
  - Binnen één werkdag
  - Binnen één tot drie werkdagen
  - Binnen langer dan drie werkdagen

1. Bestaat er voor uw ziekenhuis een **procedure** (bijvoorbeeld een klinisch zorgpad) tussen geriatrie en spoedgevallen?

- Neen, er bestaat geen procedure
- Ja, er bestaat een formele, uitgeschreven procedure (gelieve dit protocol mee te sturen)
- Ja, er bestaat een informele procedure die niet uitgeschreven is
- Momenteel bestaat er geen procedure, maar er zijn plannen voor de toekomst om dit uit te werken

1. Beschikt uw **ziekenhuis** over een intern liaison team geriatrie*?

- Ja
- Neen, ga dan onmiddellijk naar vraag 14

* De term intern geriatrisch liaison team heeft ook synoniemen bijvoorbeeld geriatrisch consultatie team, geriatrisch support team, … In het verder verloop van deze vragenlijst wordt de benaming intern liaison team geriatrie afgekort naar ILT geriatrie.

1. Gelieve in onderstaande tabel per stelling de beschikbaarheid **van het ILT geriatrie** op de dienst spoedgevallen voor de verschillende tijdsmomenten (dag, nacht, weekend) aan te duiden

|  | Dag | Nacht | Weekend |
| --- | --- | --- | --- |
| Een **medewerker van het intern liaisonteam geriatrie** is telefonisch consulteerbaar voor de oudere patiënt op spoedgevallen. | - Ja - Neen | - Ja - Neen | - Ja - Neen |
| Een **medewerker van het intern liaisonteam geriatrie** is na telefonisch contact beschikbaar aan het bed van de oudere patiënt op spoedgevallen voor specifieke casussen | - Ja - Neen | - Ja - Neen | - Ja - Neen |
| Een **medewerker van het intern liaisonteam geriatrie** komt na telefonisch contact systematisch aan het bed van de oudere patiënt op spoedgevallen voor alle casussen. | - Ja - Neen | - Ja - Neen | - Ja - Neen |
| Een **medewerker van het intern liaisonteam geriatrie** is op afgesproken momenten aanwezig op spoedgevallen. | - Ja - Neen | - Ja - Neen | - Ja - Neen |
| **Een medewerker van het intern liaisonteam geriatrie** is continu aanwezig op spoedgevallen | - Ja - Neen | - Ja - Neen | - Ja - Neen |

Indien overal neen geantwoord, ga naar vraag 14

1. In welke situaties wordt voornamelijk gebruik gemaakt van h**et ILT geriatrie** op de dienst spoedgevallen? Gelieve hieronder de 3 meest voorkomende situaties in uw ziekenhuis aan te duiden.

- Twijfel over de nood aan hospitalisatie
- Vraag op-/overname geriatrie
- Functionele problematiek
- Cognitieve problematiek
- Nood aan medische expertise
- Nood aan verdere diagnostiek
- Nood aan oppuntstelling van de sociale situatie (evaluatie en aanpassing van de thuiszorg)
- Andere, specificeer: …..

1. Hoeveel keer per maand wordt het **ILT geriatrie** geconsulteerd door de dienst spoedgevallen? …. Oproepen/ maand
2. Bent u tevreden over de ondersteuning van **het ILT geriatrie** op de dienst spoedgevallen?

○ Helemaal niet tevreden ○ Eerder niet tevreden ○ Eerder tevreden ○ Zeer tevreden

1. Beoordeel volgende stelling: De bestaande werking van het **ILT geriatrie** op spoedgevallen moet verder uitgebreid worden.

○ Helemaal niet akkoord ○ Eerder niet akkoord ○ Eerder akkoord ○ Helemaal akkoord

1. Beoordeel volgende stelling: Het **ILT geriatrie** is een meerwaarde op de dienst spoedgevallen.

○ Helemaal niet akkoord ○ Eerder niet akkoord ○ Eerder akkoord ○ Helemaal akkoord

1. Beoordeel volgende stelling: Het **ILT geriatrie** zou een meerwaarde zijn op de dienst spoedgevallen. (enkel in te vullen indien neen op vraag 8 en 9)

○ Helemaal niet akkoord ○ Eerder niet akkoord ○ Eerder akkoord ○ Helemaal akkoord

1. Beoordeel volgende stelling: De fysieke beschikbaarheid van een erkend specialist **geriatrie** op de dienst spoedgevallen is een meerwaarde.

○ Helemaal niet akkoord ○ Eerder niet akkoord ○ Eerder akkoord ○ Helemaal akkoord

1. Gelieve in onderstaande tabel elke stelling voor de verschillende tijdsmomenten (dag, nacht, weekend) te beoordelen.

|  | Dag | Nacht | Weekend |
| --- | --- | --- | --- |
| Bij opname op de spoedgevallen van uw ziekenhuis wordt doorgaans een **urgentie triage** toegepast die internationaal erkend is (vb Manchester, ESI,...) | - Ja - Neen | - Ja - Neen | - Ja - Neen |
| Tijdens de opname op de spoedgevallen van uw ziekenhuis wordt voor ouderen een **screeningsinstrument** gebruikt om patiënten met een geriatrisch risicoprofiel in kaart te brengen (vb ISAR, VIP, TRST,...) (indien neen, ga naar vraag 21) | - Ja - Neen | - Ja - Neen | - Ja - Neen |

1. Welk screeningsinstrument wordt gebruikt om patiënten met een geriatrisch risicoprofiel in kaart te brengen op de spoedgevallen van uw ziekenhuis

- Niet huisgemaakt screeningsinstrument: …..
- Huisgemaakt screeningsinstrument: …

1. Welke Cutoff score wordt gehanteerd? …..
2. Wie vult het screeningsinstrument om patiënten met een geriatrisch risicoprofiel in kaart te brengen in op spoedgevallen van uw ziekenhuis?

- Spoedgevallen verpleegkundige
- Urgentie arts
- Intern geriatrisch liaison team
- Andere, namelijk:

1. Wordt het resultaat van het screeningsinstrument om patiënten met een geriatrisch risicoprofiel in kaart te brengen op de spoedgevallen genoteerd in het patiëntendossier?

- Ja, altijd
- Ja, soms
- Neen

1. Worden de resultaten van het screeningsinstrument om patiënten met een geriatrisch risicoprofiel in kaart te brengen gebruikt in het kader van de verdere behandeling (zowel tijdens als na het verblijf op spoedgevallen) van de patiënt?

- Neen, de resultaten worden niet verder gebruikt
- Ja, de resultaten (meerdere antwoord categorieën zijn mogelijk):
  - Ondersteunen de zorgverleners op spoedgevallen in de beslissing om al dan niet het intern geriatrisch liaison team in te schakelen
  - Ondersteunen de zorgverleners op spoedgevallen in de beslissing om al dan niet een geriater in te schakelen
- Ondersteunen de zorgverleners op spoedgevallen in de beslissing om een patiënt al dan niet op de dienst geriatrie te hospitaliseren
- Ondersteunen de zorgverleners op spoedgevallen in de beslissing om de patiënt al dan niet te hospitaliseren of naar huis te ontslaan
- Andere, specificeer: ………………………………………………………

1. Moet een patiënt met een geriatrisch risicoprofiel (positieve screening), die na diagnosestelling en behandeling op spoedgevallen terug naar huis ontslagen wordt, voor ontslag systematisch gezien worden / doorverwezen worden naar een medewerker van **de dienst geriatrie?**

- Neen
- Ja

1. Zijn er op uw dienst spoedgevallen verpleegkundigen werkzaam met een **bijzondere beroepstitel of bijzondere beroepsbekwaamheid** geriatrie?

- Ja, de verhouding is … FTE van de … FTE verpleegkundigen die op spoedgevallen werken
- Neen, ga verder naar vraag 25

1. Nemen deze verpleegkundigen specifieke taken op ten aanzien van ouderen?

- Neen
- Ja, specifieer …..

1. Beschikt de dienst spoedgevallen over **referentieverpleegkundigen*** geriatrie?

- Neen ga verder naar vraag 27
- Ja: ….. verpleegkundigen

*def referentieverpleegkundige geriatrie: is een verpleegkundige aangeduid door de afdeling die beschikt over een opleiding en/of een bijzondere ervaring in de geriatrische zorg.

1. Nemen deze **referentieverpleegkundigen** specifieke taken op ten aanzien van ouderen?

- Neen
- Ja, specifieer …..

1. Werd er in het afgelopen jaar binnen de tweedaagse **verplichte** opleiding voor spoedgevallenverpleegkundigen een component geriatrie aan bod gebracht?

- Neen
- Ja
  - Geef de grote thema’s weer: …..

1. Beoordeel volgende stelling: Er moet meer geïnvesteerd worden in de opleiding van verpleegkundigen op spoedgevallen met betrekking tot zorg voor ouderen.

○ Helemaal niet akkoord ○ Eerder niet akkoord ○ Eerder akkoord ○ Helemaal akkoord

1. Beschikt de dienst spoedgevallen over aangepaste infrastructuur voor ouderen? (bv. Speciale bedden, speciale boxen, toilet, toiletverhoog, …)

- Neen
- Ja, specifieer….

1. Beoordeel volgende stelling: Op de dienst spoedgevallen is de infrastructuur voldoende uitgerust voor de kwaliteitsvolle opvang van ouderen. Vb architectuur, hulpmiddelen

○ Helemaal niet akkoord ○ Eerder niet akkoord ○ Eerder akkoord ○ Helemaal akkoord

1. Beschikt de dienst spoedgevallen van uw ziekenhuis over specifieke procedures voor de zorg voor ouderen? (vb kortere wachttijden, hygiënische maatregelen, voedingsmaatregelen, …)

- Neen
- Ja specifieer ….(indien hier protocollen of afspraken op papier rond bestaan, gelieve deze mee te sturen of licht hier kort toe)

1. Beoordeel volgende stelling: Op mijn dienst spoedgevallen zijn er voldoende specifieke procedures voor de kwaliteitsvolle opvang van ouderen

○ Helemaal niet akkoord ○ Eerder niet akkoord ○ Eerder akkoord ○ Helemaal akkoord

1. Heeft uw ziekenhuis reeds speciale initiatieven die hierboven nog niet vermeld werden, genomen om de zorg voor ouderen op spoedgevallen te verbeteren?

- Neen
- Ja, specifieer

1. In welke mate is de zorg voor ouderen op uw spoedgevallendienst aangepast aan de specifieke zorgnoden van ouderen? Beoordeel op een schaal van 1 tot 10 (1 = slecht, 10 = zeer goed).

○ 0 ○1 ○2 ○3 ○4 ○5 ○6 ○7 ○8 ○9 ○10

**Deel 2 Algemene ziekenhuisgegevens**

1. Wat is de naam van uw (fusie)ziekenhuis? …….
2. Wat is het erkenningsnummer van uw (fusie)ziekenhuis? …..
3. Gelieve onderstaande gegevens voor uw ziekenhuis in te vullen over **alle patiënten van 75 jaar of ouder** voor het meest recente jaartal waarvoor de gegevens beschikbaar zijn

| Kruis het jaartal aan waarop onderstaande gegevens betrekking hebben  ○ 2010 ○ 2011 ○ 2012 | Aantal in absolute cijfers |
| --- | --- |
| Totaal **aantal contacten** van 75 jaar of ouder die zich aanmeldden op spoedgevallen |  |
| Totaal **aantal van deze contacten** van 75 jaar of ouder die werden opgenomen op een niet-geriatrische dienst in het ziekenhuis (inclusief patiënten opgenomen op observatie- eenheid spoedgevallen) (met zelfde erkenningsnummer, dit kan eventueel ook op een andere campus) |  |
| Totaal **aantal van deze contacten** van 75 jaar of ouder die werden opgenomen op een dienst geriatrie in het ziekenhuis (met zelfde erkenningsnummer, dit kan eventueel ook op een andere campus) |  |
| Totaal **aantal van deze contacten** van 75 jaar of ouder die werden ontslagen naar de plaats van herkomst (thuis, WZC of andere) |  |
| Totaal **aantal van deze contacten** van 75 jaar of ouder die werden ontslagen naar een “ander” ziekenhuis (ander erkenningsnummer) |  |
| Totaal aantal **heropnames (= heropnames binnen de 72h)** van patiënten van 75 jaar of ouder op spoedgevallen |  |

1. Gelieve onderstaande tabel voor uw ziekenhuis aan te vullen. Er is in de tabel één lijn per campus voorzien. Met andere woorden indien uw ziekenhuis slechts over 1 campus beschikt dient u slechts één lijn in te vullen, zijn er meerdere campussen dan vult u een evenredig aantal lijnen in.

| Naam campus | Spoedgevallendienst op de campus | Aantal benutte G-bedden op de campus* | Dagziekenhuis geriatrie op de campus | Intern geriatrisch liaisonteam op de campus |
| --- | --- | --- | --- | --- |
|  | - Ja, dienst spoedgevallen met functie ‘gespecialiseerde spoedgevallenzorg’ - Ja, dienst spoedgevallen met functie ‘eerste opvang spoedgevallen’ - Neen |  | - Ja - Neen | - Ja - Neen |
|  | - Ja, dienst spoedgevallen met functie ‘gespecialiseerde spoedgevallenzorg’ - Ja, dienst spoedgevallen met functie ‘eerste opvang spoedgevallen’ - Neen |  | - Ja - Neen | - Ja - Neen |

* def benutte G-bedden: hiermee bedoelen we het aantal G-bedden die momenteel gebruikt worden voor de hospitalisatie van geriatrische patiënten. Dit aantal kan overeen komen met het aantal G-bedden waarvoor u ziekenhuis een erkenning heeft, maar kan ook een ander getal zijn indien niet alle erkende bedden gebruikt worden.

1. Gelieve in onderstaande tabel per stelling de beschikbaarheid van **een erkend specialist geriatrie (geen ASO)** op de dienst spoedgevallen voor de verschillende tijdsmomenten (dag, nacht, weekend) aan te duiden.

|  | Dag | Nacht | Weekend |
| --- | --- | --- | --- |
| Een **geriater** is telefonisch consulteerbaar voor de oudere patiënt op spoedgevallen | - Ja - Neen | - Ja - Neen | - Ja - Neen |
| Een **geriater** is na telefonisch contact beschikbaar aan het bed van de oudere patiënt op spoedgevallen voor specifieke casussen | - Ja - Neen | - Ja - Neen | - Ja - Neen |
| Een **geriater** komt na telefonisch contact systematisch aan het bed van de oudere patiënt op spoedgevallen voor alle casussen. | - Ja - Neen | - Ja - Neen | - Ja - Neen |
| Een **geriater** is op afgesproken momenten aanwezig op spoedgevallen | - Ja - Neen | - Ja - Neen | - Ja - Neen |
| Een **geriater** is continu aanwezig op spoedgevallen | - Ja - Neen | - Ja - Neen | - Ja - Neen |

1. Welke **discipline** neemt doorgaans de **finale beslissing** om een oudere patiënt van op spoedgevallen te **hospitaliseren op de dienst geriatrie**?

- De geriater
- De urgentiearts
- Beide partijen na overleg
- Andere, specificeer: …..

1. Gelieve in onderstaande tabel per stelling de beschikbaarheid van **de sociale dienst** op de dienst spoedgevallen voor de verschillende tijdsmomenten (dag, nacht, weekend) aan te duiden.

|  | Dag | Nacht | Weekend |
| --- | --- | --- | --- |
| De **sociale dienst** is telefonisch consulteerbaar voor de oudere patiënt op spoedgevallen | - Ja - Neen | - Ja   Neen | - Ja   Neen |
| De **sociale dienst** is na telefonisch contact beschikbaar aan het bed van de oudere patiënt op spoedgevallen voor specifieke casussen | - Ja - Neen | - Ja - Neen | - Ja - Neen |
| De **sociale dienst** komt na telefonisch contact systematisch aan het bed van de oudere patiënt op spoedgevallen voor alle casussen. | - Ja - Neen | - Ja - Neen | - Ja - Neen |
| De **sociale dienst** is op afgesproken momenten aanwezig op spoedgevallen | - Ja - Neen | - Ja - Neen | - Ja - Neen |
| De **sociale dienst** is continu aanwezig op spoedgevallen | - Ja - Neen | - Ja - Neen | - Ja - Neen |

1. Gelieve hieronder eventuele aanvullende opmerkingen/ suggesties over de zorg voor ouderen op spoedgevallen te noteren
2. Indien we bijkomende vragen hebben, op welk telefoonnummer of e-mailadres kunnen we u het beste bereiken?

Naam: …

Telefoonnummer: …

E-mail adres: …

1. Gelieve hier uw bankrekeningnummer achter te laten

……………………………………………………..

Naam: …………………………

**Hartelijk dank voor uw waardevolle bijdrage!**
