# Supplementary material for: Geriatric support in the emergency department: a national survey in Belgium
Source: BMC Geriatr. 2017 Mar 16;17:68. doi: 10.1186/s12877-017-0458-8 (PMC5356306; doi:10.1186/s12877-017-0458-8)
Supplement: Additional file 2: — Questionnaire geriatric support in the emergency department: French version of the questionnaire for geriatric department and emergency department. (DOCX 44 kb) [file 12877_2017_458_MOESM2_ESM.docx]

**Additional file 2: Questionnaire geriatric support in the emergency department :French version of the questionnaire for geriatric department and emergency department**

Soins gériatriques pour les personnes âgées en salle d’urgence: une étude nationale (chef de service gériatrie)

**Part 1:**

*Questions relatives aux soins des patients âgés en salle d'urgences*

1. Il existe des conventions formelles* entre les médecins traitants de la région et l’hôpital concernant les personnes âgées adressées via le/au service des urgences.

- Oui (Veuillez télécharger ici le protocole des conventions formelles )
- Non

Info: *Définition de 'convention formelle': convention écrite reconnue par les deux parties (hôpitaux et médecins généralistes) et communiquée à l’ensemble des cercles de médecins généralistes de la région.

1. En ce qui concerne dix patients âgés récemment accueillis dans le service des urgences, veuillez préciser la manière dont ils ont été adressés. De quelle manière les dix derniers patients de 75 ans ou plus ont-ils été adressés dans le service des urgences ?

Info: Prenez comme référence un jour de semaine ordinaire

Info: Il existe plusieurs catégories de réponses possibles. Veuillez indiquer pour chaque catégorie de réponse le nombre correspondant de patient (0 = aucun patient)

- Admission spontanée: …patients
- Service 100: … patient
- Lettre d'admission: …patients
- Contact téléphonique avec le médecin urgentiste ou le gériatre: …patients
- Autre: …patients, spécifiez….

1. Quel type de procédure préférez-vous pour l’admission d’une personne âgée via le service des urgences ?

- Contact informel: lettre d'admission
- Contact informel: contact téléphonique avec le gériatre ou le médecin urgentiste
- Contact informel: lettre d'admission en combinaison avec un contact téléphonique
- Accord formel au sein de la région

1. Votre hôpital dispose-t-il d’un hôpital de jour gériatrique ?

- Oui
- Non, allez alors immédiatement à la question 6

1. Un rendez-vous urgent peut-il être pris par le service des urgences à l’hôpital de jour gériatrique afin d’éviter une hospitalisation ?

- Non ceci est impossible
- Oui ceci est possible, dans un délai de un jour ouvrable
- Oui ceci est possible, dans un délai de deux à trois jours ouvrables
- Oui ceci est possible, dans un délai supérieur à trois jours ouvrables

1. Existe-t-il dans votre hôpital une procédure (par exemple un trajet de soins) entre la gériatrie et le service des urgences?

- Non, une telle procédure n'existe pas
- Oui, il existe une procédure formelle écrite (Veuillez télécharger le protocole de la procédure entre le service des urgences et le service de gériatrie)
- Oui, il existe une procédure informelle qui n'est pas écrite
- Il n'existe actuellement aucune procédure mais des projets sont en cours pour la mettre en œuvre

1. Existe-t-il dans votre hôpital une équipe de gériatrie de liaison interne*?

- Oui
- Non, allez alors immédiatement à la question 14

*Info: Le terme équipe de gériatrie de liaison interne porte également d’autres noms, synonymes, tels que équipe de consultation gériatrique, équipe de soutien gériatrique, … Dans la suite de ce questionnaire, le titre équipe de gériatrie de liaison interne sera abrégé EGL.

1. Veuillez indiquer dans le tableau ci-dessous la disponibilité de l’EGL pour le service des urgences aux différents moments de la journée (jour, nuit, weekend).

|  | Jour | Nuit | Weekend |
| --- | --- | --- | --- |
| Un collaborateur de l'EGL est consultable par téléphone au sujet d'un patient âgé présent aux urgences | - oui - non | - oui - non | - oui - non |
| Un collaborateur de l'EGL est disponible après contact téléphonique au lit du patient âgé aux urgences pour des situations spécifiques | - oui - non | - oui - non | - oui - non |
| Un collaborateur de l'EGL vient systématiquement après contact téléphonique au lit du patient âgé admis aux urgences et ce pour chaque cas | - oui - non | - oui - non | - oui - non |
| Un collaborateur de l'EGL est présent à des moments convenus aux urgences | - oui - non | - oui - non | - oui - non |
| Un collaborateur de l'EGL est présent en permanence aux urgences | - oui - non | - oui - non | - oui - non |

Si vous avez répondu non à chaque question, allez à la question 14

1. Dans quelles situations fait-on principalement usage de l’**EGL** au service des urgences ? Veuillez indiquer les trois situations les plus fréquemment rencontrées dans votre hôpital.

- Doute quant à la nécessité d’une hospitalisation
- Demande d’admission ou de transfert en gériatrie
- Problématique fonctionnelle
- Problématique cognitive
- Besoin d’expertise médicale
- Besoin d’un diagnostic complémentaire
- Besoin d’un bilan de la situation sociale (évaluation et adaptation des soins à domicile)
- Autre, spécifiez : …..

1. Combien de fois par mois l’EGL est-elle appelée/consultée par le service des urgences?

…. Appels par mois

1. L’EGL qui intervient en salle d’urgence va:

- Uniquement apporter une réponse à la question posée
- Réaliser une évaluation gériatrique de base adaptée à un service de soins d'urgence
- Réaliser une évaluation gériatrique standardisée au sein du service des urgences comme dans les autres services d'hospitalisation

1. Evaluez l’affirmation suivante: 'Le fonctionnement actuel de l’EGL au sein du service des urgences devrait être plus développé'.

- Pas du tout d'accord
- Plutôt pas d'accord
- Plutôt d'accord
- Tout à fait d'accord

1. Evaluez l’affirmation suivante: 'L’EGL constitue une plus-value pour le service des urgences'.

- Pas du tout d'accord
- Plutôt pas d'accord
- Plutôt d'accord
- Tout à fait d'accord

1. Evaluez l’affirmation suivante : **L’EGL** constituerait une plus-value pour le service des urgences. (A ne remplir que si la réponse est non aux questions 7 ou 8)

- Pas du tout d'accord
- Plutôt pas d'accord
- Plutôt d'accord
- Tout à fait d'accord

1. Evaluez l’affirmation suivante : La disponibilité physique d’un médecin spécialiste reconnu en **gériatrie** au sein du service des urgences constitue une plus-value.

- Pas du tout d'accord
- Plutôt pas d'accord
- Plutôt d'accord
- Tout à fait d'accord

1. Veuillez évaluer, dans le tableau suivant, chaque affirmation concernant les différents moments de la journée (jour, nuit, week-end).

|  | Jour | Nuit | Weekend |
| --- | --- | --- | --- |
| A l'admission au service des urgences de votre hôpital, on applique systématiquement un triage d'urgence internationalement reconnu (p.ex. Manchester, ESI,...) | - Oui - Non | - Oui - Non | - Oui - Non |
| En cours de séjour au service des urgences de votre hôpital, on utilise un instrument de dépistage afin d'identifier les patients à profil de risque gériatrique (p.ex. ISAR, VIP, TRST,...) (si non, allez à la question 22) | - Oui - Non | - Oui - Non | - Oui - Non |

1. Quel instrument de dépistage utilisez-vous pour identifier les patients à profil de risque gériatrique au sein du service des urgences de votre hôpital?

Info: Veuillez indiquer une réponse et inscrire le nom de l’instrument de dépistage dans l’espace prévu

- Un instrument non développé par vos soins:…..
- Un instrument développé par vos: …

1. Quel est le cut-off utilisé?....
2. Qui remplit le plus souvent l’instrument d’identification des patients à profil de risque gériatrique en salle d’urgence de votre hôpital?

- Un(e) infirmier(e) du service des urgences
- Un médecin urgentiste
- L’équipe de gériatrie de liaison interne (EGL)
- Autres, précisez : …

1. Le résultat du dépistage en salle d’urgence des patients à profil de risque gériatrique est-il notifié dans le dossier du patient?

- Oui, toujours
- Oui, parfois
- Non

1. Les résultats de dépistage du profil de risque gériatrique sont-ils utilisés dans le cadre de la poursuite du traitement du patient (à la fois pendant et après le séjour dans le service des urgences) ?

- Non, les résultats ne sont pas utilisés ultérieurement
- Oui, les résultats (plusieurs réponses sont possibles) :
  - Aident les soignants en salle d’urgence à décider de faire intervenir ou non l’équipe de gériatrie de liaison interne
  - Aident les soignants en salle d’urgence à décider de faire intervenir ou non un gériatre
  - Aident les soignants en salle d’urgence à décider d’hospitaliser ou non un patient en gériatrie
  - Aident les soignants en salle d’urgence à décider d’hospitaliser le patient ou de le laisser retourner à domicile
  - Autre, spécifiez : ……………

1. Un patient avec profil de risque gériatrique (dépistage positif) qui, après diagnostic et traitement en salle d’urgence, est renvoyé à domicile, doit-il avant son départ être vu et/ou adressé à un membre du **service de gériatrie** ?

- Non
- Oui

1. Y a-t-il eu durant l’année 2012-2013 des initiatives de formation émanant du service de gériatrie et destinées spécifiquement au service des urgences?

- Non
- Oui

1. Spécifiez le nombre total d’heures des initiatives de formation: …
2. Spécifiez la date de la dernière initiative de formation:….
3. Spécifiez le sujet de la dernière initiative de formation:
4. Evaluez l’affirmation suivante: Le service des urgences dispose d’une infrastructure suffisamment équipée pour une prise en charge de qualité des personnes âgées. (p. ex. l'architecture, des aides technique,...)

- Pas du tout d'accord
- Plutôt pas d'accord
- Plutôt d'accord
- Tout à fait d'accord

1. Votre hôpital a-t-il déjà pris des initiatives particulières qui n’ont pas été décrites ci-dessus afin d’améliorer la prise en charge des personnes âgées aux urgences ?

- Non
- Oui, spécifiez : …

1. Dans quelle mesure la prise en charge des personnes âgées au service des urgences est-elle adaptée aux besoins spécifiques des personnes âgées ? Evaluez sur une échelle de 1 à 10 (1 = mauvais, 10 = optimal).

○ 0 ○1 ○2 ○3 ○4 ○5 ○6 ○7 ○8 ○9 ○10

**Partie 2 Données générales relatives à l’hôpital**

1. Quel est le nom de votre hôpital (éventuellement fusionné) ?.....
2. Quel est le numéro d’agrément de votre hôpital (éventuellement fusionné) ?
3. Veuillez compléter le tableau ci-dessous concernant votre hôpital. Celui-ci comporte une ligne par campus. En d’autres termes, si votre hôpital ne comporte qu’un campus, il ne vous faudra remplir qu’une seule ligne, s’il y en a plusieurs, il vous faudra remplir le nombre de lignes correspondant au nombre de campus

| Nom du campus | Service des urgences sur le campus | Nombre total de lits-G utilisés sur le campus* | Hôpital de jour gériatrique sur le campus | Equipe de gériatrie de liaison interne sur le campus |
| --- | --- | --- | --- | --- |
|  | - Oui, service des urgences avec fonction « soins urgents spécialisés » - Oui, service des urgences avec fonction « soins urgents » - Non |  | - Oui - Non | - Oui - Non |
| Veuillez compléter de la même manière les données concernant tous les campus/sites sur le site web. | | | | |

*Définition lits-G utilisés : nous entendons par là le nombre de lits-G actuellement en fonction pour l’hospitalisation des patients gériatriques. Ceci peut correspondre au nombre total de lits-G pour lequel votre hôpital possède un agrément mais peut aussi être différent si tous les lits reconnus ne sont pas utilisés.

1. Veuillez remplir le tableau ci-dessous en rapport avec votre hôpital.

*Une ligne est attribuée à chaque gériatre. Par exemple, s’il y a trois gériatres en fonction dans votre hôpital, veuillez remplir trois lignes.

*Veuillez indiquer un nombre entre 0 et 10 concernant l’affectation totale et l'affectation au service de gériatrie/des urgences. Veuillez indiquer « oui » ou « non » concernant le fonctionnement sur le campus avec le service des urgences.

| Gériatre | Total sous contrat à l'Hôpital (en 10ème ETP, exceptés les 10ème "ETP académiques") (.../10) | Affecté au service de gériatrie (en 10ième ETP du total) (.../10) | En fonction où ce trouve le service des urgences (oui/non) | Presté au service des urgences (en 10ième ETP total, proportion du total) (.../10) |
| --- | --- | --- | --- | --- |
| Gériatre | …/10 | …/10 | - Oui - Non | …/10 |
| Veuillez compléter de la même manière les données concernant tous les gériatres sur le site web | | | | |

1. Veuillez indiquer dans le tableau ci-dessous la disponibilité **d’un spécialiste reconnu** **en gériatrie (pas un assistant en formation)** pour service des urgences aux différents moments de la journée (jour, nuit, week-end).

|  | Jour | Nuit | Week-end |
| --- | --- | --- | --- |
| Un **gériatre** est consultable par téléphone au sujet d’un patient âgé présent aux urgences | - Oui - Non | - Oui - Non | - Oui - Non |
| Un **gériatre** est disponible après contact téléphonique au lit du patient âgé présent aux urgences pour des situations spécifiques | - Oui - Non | - Oui - Non | - Oui - Non |
| Un **gériatre** vient systématiquement après contact téléphonique au lit du patient âgé aux urgences et ce chaque cas | - Oui - Non | - Oui - Non | - Oui - Non |
| Un **gériatre** est présent à des moments convenus aux urgences | - Oui - Non | - Oui - Non | - Oui - Non |
| Un **gériatre** est présent en permanence aux urgences | - Oui - Non | - Oui - Non | - Oui - Non |

1. Quelle **discipline** prend habituellement **la décision finale** quant à **l’hospitalisation dans le service de gériatrie** d’un patient âgé présent aux urgences ?

- Le gériatre
- Le médecin urgentiste
- Les deux après discussion
- Autre, spécifiez : …..

1. Veuillez indiquer dans le tableau ci-dessous la disponibilité du **service social** pour le service des urgences aux différents moments de la journée (jour, nuit, week-end).

|  | Jour | Nuit | Week-end |
| --- | --- | --- | --- |
| Le **service social** est consultable par téléphone au sujet d’un patient âgé présent aux urgences | - Oui - Non | - Oui - Non | - Oui - Non |
| Le **service social** est disponible après contact téléphonique au lit du patient âgé présent aux urgences pour des situations spécifiques | - Oui - Non | - Oui - Non | - Oui - Non |
| Le **service social** vient systématiquement après contact téléphonique au lit du patient âgé présent aux urgences et ce pour chaque cas | - Oui - Non | - Oui - Non | - Oui - Non |
| Le **service social** est présent à des moments convenus aux urgences | - Oui - Non | - Oui - Non | - Oui - Non |
| Le **service social** est présent en permanence aux urgences | - Oui - Non | - Oui - Non | - Oui - Non |

1. Veuillez indiquer ci-dessous vos commentaires/suggestions supplémentaires éventuelles concernant la prise en charge des personnes âgées en salle d’urgences.
2. Si nous avons des questions complémentaires, à quel numéro de téléphone ou adresse électronique pourrions-nous vous contacter ?

Nom : …

Numéro de téléphone : …

Adresse email : …

1. Veuillez indiquer ci-dessous votre numéro de compte bancaire

……………………………………………………..

Nom : ……………………………………………..

**Nous vous remercions pour votre précieuse contribution !**

Soins gériatriques pour les personnes âgées en salle d’urgence: une étude nationale (chef de service des urgence)

**Part 1:**

*Questions relatives aux soins des patients âgés en salle d'urgences*

1. Il existe des conventions formelles* entre les médecins traitants de la région et l’hôpital concernant les personnes âgées adressées via le/au service des urgences.

- Oui (Veuillez télécharger ici le protocole des conventions formelles )
- Non

Info: *Définition de 'convention formelle': convention écrite reconnue par les deux parties (hôpitaux et médecins généralistes) et communiquée à l’ensemble des cercles de médecins généralistes de la région.

1. En ce qui concerne dix patients âgés récemment accueillis dans le service des urgences, veuillez préciser la manière dont ils ont été adressés. De quelle manière les dix derniers patients de 75 ans ou plus ont-ils été adressés dans le service des urgences ?

Info: Prenez comme référence un jour de semaine ordinaire

Info: Il existe plusieurs catégories de réponses possibles. Veuillez indiquer pour chaque catégorie de réponse le nombre correspondant de patient (0 = aucun patient)

- Admission spontanée: …patients
- Service 100: … patient
- Lettre d'admission: …patients
- Contact téléphonique avec le médecin urgentiste ou le gériatre: …patients
- Autre: …patients, spécifiez….

1. Quel type de procédure préférez-vous pour l’admission d’une personne âgée via le service des urgences ?

- Contact informel: lettre d'admission
- Contact informel: contact téléphonique avec le gériatre ou le médecin urgentiste
- Contact informel: lettre d'admission en combinaison avec un contact téléphonique
- Accord formel au sein de la région

1. Votre hôpital dispose-t-il d’un hôpital de jour gériatrique ?

- Oui
- Non, allez alors immédiatement à la question 6

1. Un rendez-vous urgent peut-il être pris par le service des urgences à l’hôpital de jour gériatrique afin d’éviter une hospitalisation ?

- Non ceci est impossible
- Oui ceci est possible, dans un délai de un jour ouvrable
- Oui ceci est possible, dans un délai de deux à trois jours ouvrables
- Oui ceci est possible, dans un délai supérieur à trois jours ouvrables

1. Existe-t-il dans votre hôpital une procédure (par exemple un trajet de soins) entre la gériatrie et le service des urgences?

- Non, une telle procédure n'existe pas
- Oui, il existe une procédure formelle écrite (Veuillez télécharger le protocole de la procédure entre le service des urgences et le service de gériatrie)
- Oui, il existe une procédure informelle qui n'est pas écrite
- Il n'existe actuellement aucune procédure mais des projets sont en cours pour la mettre en œuvre

1. Existe-t-il dans votre hôpital une équipe de gériatrie de liaison interne*?

- Oui
- Non, allez alors immédiatement à la question 14

*Info: Le terme équipe de gériatrie de liaison interne porte également d’autres noms, synonymes, tels que équipe de consultation gériatrique, équipe de soutien gériatrique, … Dans la suite de ce questionnaire, le titre équipe de gériatrie de liaison interne sera abrégé EGL.

1. Veuillez indiquer dans le tableau ci-dessous la disponibilité de l’EGL pour le service des urgences aux différents moments de la journée (jour, nuit, weekend).

|  | Jour | Nuit | Weekend |
| --- | --- | --- | --- |
| Un collaborateur de l'EGL est consultable par téléphone au sujet d'un patient âgé présent aux urgences | - oui - non | - oui - non | - oui - non |
| Un collaborateur de l'EGL est disponible après contact téléphonique au lit du patient âgé aux urgences pour des situations spécifiques | - oui - non | - oui - non | - oui - non |
| Un collaborateur de l'EGL vient systématiquement après contact téléphonique au lit du patient âgé admis aux urgences et ce pour chaque cas | - oui - non | - oui - non | - oui - non |
| Un collaborateur de l'EGL est présent à des moments convenus aux urgences | - oui - non | - oui - non | - oui - non |
| Un collaborateur de l'EGL est présent en permanence aux urgences | - oui - non | - oui - non | - oui - non |

Si vous avez répondu non à chaque question, allez à la question 14

1. Dans quelles situations fait-on principalement usage de l’**EGL** au service des urgences ? Veuillez indiquer les trois situations les plus fréquemment rencontrées dans votre hôpital.

- Doute quant à la nécessité d’une hospitalisation
- Demande d’admission ou de transfert en gériatrie
- Problématique fonctionnelle
- Problématique cognitive
- Besoin d’expertise médicale
- Besoin d’un diagnostic complémentaire
- Besoin d’un bilan de la situation sociale (évaluation et adaptation des soins à domicile)
- Autre, spécifiez : …..

1. Combien de fois par mois l’EGL est-elle appelée/consultée par le service des urgences?

…. Appels par mois

1. Etes-vous satisfait du soutien fourni par l’EGL au service des urgences?

- Pas du tout satisfait
- Plutôt insatisfait
- Plutôt satisfait
- Tout à fait satisfait

1. Evaluez l’affirmation suivante: 'Le fonctionnement actuel de l’EGL au sein du service des urgences devrait être plus développé'.

- Pas du tout d'accord
- Plutôt pas d'accord
- Plutôt d'accord
- Tout à fait d'accord

1. Evaluez l’affirmation suivante: 'L’EGL constitue une plus-value pour le service des urgences'.

- Pas du tout d'accord
- Plutôt pas d'accord
- Plutôt d'accord
- Tout à fait d'accord

1. Evaluez l’affirmation suivante : **L’EGL** constituerait une plus-value pour le service des urgences. (A ne remplir que si la réponse est non aux questions 8 ou 9)

- Pas du tout d'accord
- Plutôt pas d'accord
- Plutôt d'accord
- Tout à fait d'accord

1. Evaluez l’affirmation suivante : La disponibilité physique d’un médecin spécialiste reconnu en **gériatrie** au sein du service des urgences constitue une plus-value.

- Pas du tout d'accord
- Plutôt pas d'accord
- Plutôt d'accord
- Tout à fait d'accord

1. Veuillez évaluer, dans le tableau suivant, chaque affirmation concernant les différents moments de la journée (jour, nuit, week-end).

|  | Jour | Nuit | Weekend |
| --- | --- | --- | --- |
| A l'admission au service des urgences de votre hôpital, on applique systématiquement un triage d'urgence internationalement reconnu (p.ex. Manchester, ESI,...) | - Oui - Non | - Oui - Non | - Oui - Non |
| En cours de séjour au service des urgences de votre hôpital, on utilise un instrument de dépistage afin d'identifier les patients à profil de risque gériatrique (p.ex. ISAR, VIP, TRST,...) (si non, allez à la question 21) | - Oui - Non | - Oui - Non | - Oui - Non |

1. Quel instrument de dépistage utilisez-vous pour identifier les patients à profil de risque gériatrique au sein du service des urgences de votre hôpital?

Info: Veuillez indiquer une réponse et inscrire le nom de l’instrument de dépistage dans l’espace prévu

- Un instrument non développé par vos soins:…..
- Un instrument développé par vos: …

1. Quel est le cut-off utilisé?....
2. Qui remplit le plus souvent l’instrument d’identification des patients à profil de risque gériatrique en salle d’urgence de votre hôpital?

- Un(e) infirmier(e) du service des urgences
- Un médecin urgentiste
- L’équipe de gériatrie de liaison interne (EGL)
- Autres, précisez : …

1. Le résultat du dépistage en salle d’urgence des patients à profil de risque gériatrique est-il notifié dans le dossier du patient?

- Oui, toujours
- Oui, parfois
- Non

1. Les résultats de dépistage du profil de risque gériatrique sont-ils utilisés dans le cadre de la poursuite du traitement du patient (à la fois pendant et après le séjour dans le service des urgences) ?

- Non, les résultats ne sont pas utilisés ultérieurement
- Oui, les résultats (plusieurs réponses sont possibles) :
  - Aident les soignants en salle d’urgence à décider de faire intervenir ou non l’équipe de gériatrie de liaison interne
  - Aident les soignants en salle d’urgence à décider de faire intervenir ou non un gériatre
  - Aident les soignants en salle d’urgence à décider d’hospitaliser ou non un patient en gériatrie
  - Aident les soignants en salle d’urgence à décider d’hospitaliser le patient ou de le laisser retourner à domicile
  - Autre, spécifiez : ……………

1. Un patient avec profil de risque gériatrique (dépistage positif) qui, après diagnostic et traitement en salle d’urgence, est renvoyé à domicile, doit-il avant son départ être vu et/ou adressé à un membre du **service de gériatrie** ?

- Non
- Oui

1. Y a-t-il dans le service des urgences des infirmiers qui disposent **d’un titre ou d’une compétence particulière en gériatrie** ?

- Oui, le rapport est de …… ETP pour …….ETP infirmier en fonction au service des urgences.
- Non : allez à la question 25

1. Est-ce que ces infirmiers assument des tâches spécifiques à l’égard des personnes âgées?

- Non
- Oui, spécifiez : ….

1. Le service des urgences dispose-t-il **d’un infirmier de référence*** en gériatrie ?

- Non, allez à la question 27
- Oui :…. infirmiers

* La définition de l’infirmier de référence en gériatrie : un infirmier attaché au service qui dispose d’une formation et/ou d’une expérience particulière dans les soins gériatriques.

1. Est-ce que ces **infirmiers de référence** assument des tâches spécifiques à l’égard des personnes âgées ?

- Non
- Oui : spécifiez ….

1. Au cours de la dernière année, a-t-on abordé un sujet de gériatrie dans le cadre des deux journées obligatoires de formation pour les infirmières du service des urgences?

- Non
- Oui
  - Donnez les grands thèmes : ….

1. Evaluez l’affirmation suivante : Il faut investir plus dans la formation des infirmiers du service des urgences concernant les soins aux personnes âgées.

- Pas du tout d'accord
- Plutôt pas d'accord
- Plutôt d'accord
- Tout à fait d'accord

1. Le service des urgences dispose-t-il d’une infrastructure adaptée aux personnes âgées (p.ex. des lits spéciaux, des box spéciaux, toilettes, rehausseurs de toilette, …) ?

- Non
- Oui, spécifiez : …

1. Evaluez l’affirmation suivante: Le service des urgences dispose d’une infrastructure suffisamment équipée pour une prise en charge de qualité des personnes âgées. (p. ex. l'architecture, des aides technique,...)

- Pas du tout d'accord
- Plutôt pas d'accord
- Plutôt d'accord
- Tout à fait d'accord

1. Le service des urgences de votre hôpital dispose-t-il de procédures spécifiques pour la prise en charge des personnes âgées (p.ex. réduction du temps d’attente, mesures d’hygiène, mesures alimentaires, …)?

Info: S’il existe des protocoles ou des conventions écrites, veuillez les décrire brièvement in ci-après

- Oui, spécifiez...
- Non

1. Evaluez l’affirmation suivante : Mon service des urgences dispose de suffisamment de procédures spécifiques pour une prise en charge de qualité des personnes âgées.

- Pas du tout d'accord
- Plutôt pas d'accord
- Plutôt d'accord
- Tout à fait d'accord

1. Votre hôpital a-t-il déjà pris des initiatives particulières qui n’ont pas été décrites ci-dessus afin d’améliorer la prise en charge des personnes âgées aux urgences ?

- Non
- Oui, spécifiez : …

1. Dans quelle mesure la prise en charge des personnes âgées au service des urgences est-elle adaptée aux besoins spécifiques des personnes âgées ? Evaluez sur une échelle de 1 à 10 (1 = mauvais, 10 = optimal).

○ 0 ○1 ○2 ○3 ○4 ○5 ○6 ○7 ○8 ○9 ○10

**Partie 2 Données générales relatives à l’hôpital**

1. Quel est le nom de votre hôpital (éventuellement fusionné) ?.....
2. Quel est le numéro d’agrément de votre hôpital (éventuellement fusionné) ?
3. Veuillez compléter les informations ci-dessous, relatives à votre hôpital, pour **tous les patients de 75 ans ou plus** concernant l’année la plus récente pour laquelle ces données sont disponibles.

| Indiquez l’année à laquelle se rapportent les données  ο 2010 ο 2011 ο 2012 | Nombre total |
| --- | --- |
| **Nombre total de contacts** patients de 75 ans ou plus admis au sein du service des urgences |  |
| **Nombre total de ces contacts** qui ont été admis dans un service non gériatrique de l’hôpital (en ce compris les patients admis en unité d’observation des urgences) (pour un même numéro d’agrément, mais peut aussi être éventuellement sur un autre campus) |  |
| **Nombre total de ces contacts** qui ont été admis dans un service de gériatrie de l’hôpital (pour un même numéro d’agrément, mais peut aussi être éventuellement sur un autre campus) |  |
| **Nombre total de ces contacts** qui sont retournés dans leur lieu de vie d’origine (domicile, MRS ou autre) |  |
| **Nombre total de ces contacts** qui ont été référés à un « autre » hôpital (autre numéro d’agrément) |  |
| Nombre total de **réadmissions** **précoces** en salle d’urgences (= **réadmission endéans les 72 heures**) de patients de 75 ans ou plus |  |

1. Veuillez compléter le tableau ci-dessous concernant votre hôpital. Celui-ci comporte une ligne par campus. En d’autres termes, si votre hôpital ne comporte qu’un campus, il ne vous faudra remplir qu’une seule ligne, s’il y en a plusieurs, il vous faudra remplir le nombre de lignes correspondant au nombre de campus

| Nom du campus | Service des urgences sur le campus | Nombre total de lits-G utilisés sur le campus* | Hôpital de jour gériatrique sur le campus | Equipe de gériatrie de liaison interne sur le campus |
| --- | --- | --- | --- | --- |
|  | - Oui, service des urgences avec fonction « soins urgents spécialisés » - Oui, service des urgences avec fonction « soins urgents » - Non |  | - Oui - Non | - Oui - Non |
| Veuillez compléter de la même manière les données concernant tous les campus/sites sur le site web. | | | | |

*Définition lits-G utilisés : nous entendons par là le nombre de lits-G actuellement en fonction pour l’hospitalisation des patients gériatriques. Ceci peut correspondre au nombre total de lits-G pour lequel votre hôpital possède un agrément mais peut aussi être différent si tous les lits reconnus ne sont pas utilisés.

1. Veuillez indiquer dans le tableau ci-dessous la disponibilité **d’un spécialiste reconnu** **en gériatrie (pas un assistant en formation)** pour service des urgences aux différents moments de la journée (jour, nuit, week-end).

|  | Jour | Nuit | Week-end |
| --- | --- | --- | --- |
| Un **gériatre** est consultable par téléphone au sujet d’un patient âgé présent aux urgences | - Oui - Non | - Oui - Non | - Oui - Non |
| Un **gériatre** est disponible après contact téléphonique au lit du patient âgé présent aux urgences pour des situations spécifiques | - Oui - Non | - Oui - Non | - Oui - Non |
| Un **gériatre** vient systématiquement après contact téléphonique au lit du patient âgé aux urgences et ce chaque cas | - Oui - Non | - Oui - Non | - Oui - Non |
| Un **gériatre** est présent à des moments convenus aux urgences | - Oui - Non | - Oui - Non | - Oui - Non |
| Un **gériatre** est présent en permanence aux urgences | - Oui - Non | - Oui - Non | - Oui - Non |

1. Quelle **discipline** prend habituellement **la décision finale** quant à **l’hospitalisation dans le service de gériatrie** d’un patient âgé présent aux urgences ?

- Le gériatre
- Le médecin urgentiste
- Les deux après discussion
- Autre, spécifiez : …..

1. Veuillez indiquer dans le tableau ci-dessous la disponibilité du **service social** pour le service des urgences aux différents moments de la journée (jour, nuit, week-end).

|  | Jour | Nuit | Week-end |
| --- | --- | --- | --- |
| Le **service social** est consultable par téléphone au sujet d’un patient âgé présent aux urgences | - Oui - Non | - Oui - Non | - Oui - Non |
| Le **service social** est disponible après contact téléphonique au lit du patient âgé présent aux urgences pour des situations spécifiques | - Oui - Non | - Oui - Non | - Oui - Non |
| Le **service social** vient systématiquement après contact téléphonique au lit du patient âgé présent aux urgences et ce pour chaque cas | - Oui - Non | - Oui - Non | - Oui - Non |
| Le **service social** est présent à des moments convenus aux urgences | - Oui - Non | - Oui - Non | - Oui - Non |
| Le **service social** est présent en permanence aux urgences | - Oui - Non | - Oui - Non | - Oui - Non |

1. Veuillez indiquer ci-dessous vos commentaires/suggestions supplémentaires éventuelles concernant la prise en charge des personnes âgées en salle d’urgences.
2. Si nous avons des questions complémentaires, à quel numéro de téléphone ou adresse électronique pourrions-nous vous contacter ?

Nom : …

Numéro de téléphone : …

Adresse email : …

1. Veuillez indiquer ci-dessous votre numéro de compte bancaire

……………………………………………………..

Nom : ……………………………………………..

**Nous vous remercions pour votre précieuse contribution !**
